# Preliminary Evidence for Sex-Specific Trends in Probiotic Modulation of Gut Saccharibacteria in Familial Mediterranean Fever Patients: Effects of *Lactobacillus acidophilus* INMIA 9602 Er 317/402 and *Escherichia coli* M-17

**DOI:** 10.3390/ijms26188959

**Published:** 2025-09-15

**Authors:** Natalya Harutyunyan, Lena Stepanyan, Marine Balayan, Anahit Manvelyan, Elya Pepoyan, Vardan Tsaturyan, Tamas Torok, Astghik Pepoyan

**Affiliations:** 1 Department of Food Safety and Biotechnology, Armenian National Agrarian University, 74 Teryan St., Yerevan 0009, Armenia; nharutyunyan@anau.am (N.H.); lstepanyan@anau.am (L.S.); mbalayan@anau.am (M.B.); elya.pepoyan@meduni.am (E.P.); 2 Military Therapy Department, Yerevan State Medical University after Mkhitar Heratsi, 2 Koryun St., Yerevan 0025, Armenia; vardan.tsaturyan@meduni.am; 3 Ecology Department, Berkeley, Lawrence Berkeley National Laboratory, Berkeley, CA 94720, USA; ttorok@lbl.gov

**Keywords:** candidate phyla radiation bacteria, *Candidatus saccharibacteria*, *Schaalia odontolytica*, familial Mediterranean fever, probiotics, Narine^®^, Colibacteron^®^

## Abstract

Candidate Phyla Radiation bacteria are emerging members of the human microbiota, particularly in oral and gut environments. Saccharibacteria were previously identified in the gut microbiota of healthy individuals and women diagnosed with familial Mediterranean fever (FMF), a monogenic autoinflammatory disorder prevalent in the eastern Mediterranean region, including Armenia. This study aimed to assess the prevalence and diversity of *Saccharibacteria* spp. and its basebiont *Schaalia odontolytica* in FMF patients, explore gender differences, and evaluate the modulation potential of two locally produced probiotics: *Lactobacillus acidophilus* INMIA9602 Er317/402 (Narine^®^, VITAMAX-E, Yerevan, Armenia) and *Escherichia coli* M-17 (Colibacteron^®^, VITAMAX-E, Yerevan, Armenia). The abundance and behavior of saccharibacteria and *S. odontolytica* appear to vary depending on health status and sex. Placebo administration caused both quantitative and qualitative shifts, suggesting a possible interaction between *Candidatus saccharibacteria* spp. and *Schaalia odontolytica*, though the underlying biological significance remains to be clarified. Narine administration appeared to increase the abundance of *Candidatus saccharibacteria* operational taxonomic units (OTUs) in FMF women and *S. odontolytica* OTUs in FMF men, whereas Colibacteron selectively decreased certain OTUs, predominantly in FMF women. These findings underscore the need to further investigate saccharibacteria’s role in systemic inflammation and probiotic-mediated modulation of the gut microbiota.

## 1. Introduction

Candidate Phyla Radiation (CPR) bacteria, including the saccharibacteria group (formerly TM7), represent a unique branch of the bacterial domain. CPR bacteria are characterized by extremely small cell sizes, streamlined genomes, and limited metabolic capacities, suggesting parasitic or symbiotic lifestyles [1,2]. They are estimated to comprise approximately 15% of total bacterial diversity, encompassing over 70 phyla. The first sequenced representative of CPR bacteria, a *Saccharibacteria* sp., was isolated from an environmental peat sample in Germany [3]. Although predominantly detected in non-host environments, CPR taxa, including *Candidatus saccharibacteria* spp., have also been observed in the human microbiome, particularly in the oral cavity [4,5,6]. In this niche, saccharibacteria live as epibionts on the surface of host bacteria (“basebiont”). These physical associations appear to be crucial for their survival [7,8]. Intriguingly, beyond their potential pathogenicity toward host bacteria, saccharibacteria may also play protective roles for the human host, potentially reducing inflammation in conditions such as periodontitis [7].

The previous investigations [9] revealed the presence of *Saccharibacteria* spp. in the gut microbiota of both healthy women and women with Familial Mediterranean Fever (FMF), a monogenic autoinflammatory disease prevalent in the Armenian population [10,11]. These findings raise important questions regarding the ecological role of saccharibacteria in the gut, especially under conditions of chronic inflammation. Furthermore, *Saccharibacteria* spp. may influence or be influenced by inert or placebo-like substances, possibly modulating host responses in unexpected ways [12,13]. This intriguing phenomenon highlights the need to consider microbial components even in studies using seemingly neutral treatments.

Probiotics, live microorganisms that confer health benefits to the host, have garnered increasing attention for their capacity to modulate the gut microbiota [14,15]. In FMF patients probiotics have been used to improve gastrointestinal function, mitigate side effects of long-term colchicine therapy, and possibly influence systemic inflammation [16,17]. The previous studies suggest that probiotics may impact both the composition and functional activity of microbial communities in the gut and oral cavity [18,19].

Thus, despite growing interest in CPR bacteria, including *Saccharibacteria* spp., their role within the human gut microbiota, particularly under conditions of chronic autoinflammatory diseases such as FMF, remains poorly defined. Most existing studies focus on oral microbiomes, leaving a gap in knowledge regarding gut-associated *Saccharibacteria* and their interactions with host bacteria. This study is among the first to explore the potential modulatory effects of probiotics on gut *Saccharibacteria* spp. and their host, *Schaalia odontolytica*, in FMF patients, with a specific focus on sex-dependent patterns. By addressing these unexplored dynamics, the study contributes to a deeper understanding of CPR taxa ecology in systemic inflammation.

## 2. Results

### 2.1. Quantitative Distribution of Gut Candidatus saccharibacteria spp. in Male FMF Patients

Baseline comparisons between healthy and FMF groups revealed no significant differences (*p* > 0.05 across all OTUs).

In healthy men baseline hybridization scores for the detected OTUs ranged from 373.53 ± 40.42 (OTU 2) to 4870.98 ± 1117.07 (OTU 1), with post-placebo values showing only minor fluctuations. Similarly, FMF males showed no significant variation after placebo administration.

No statistically significant changes were observed in the hybridization scores of gut *Candidatus saccharibacteria* spp. before and after placebo administration in either healthy or FMF-affected males (Table 1, *p* > 0.05 for all).

### 2.2. Effects of Probiotics Narine and Colibacteron on the Gut Candidatus saccharibacteria spp. Distribution in FMF Patients

**Figure 1:**
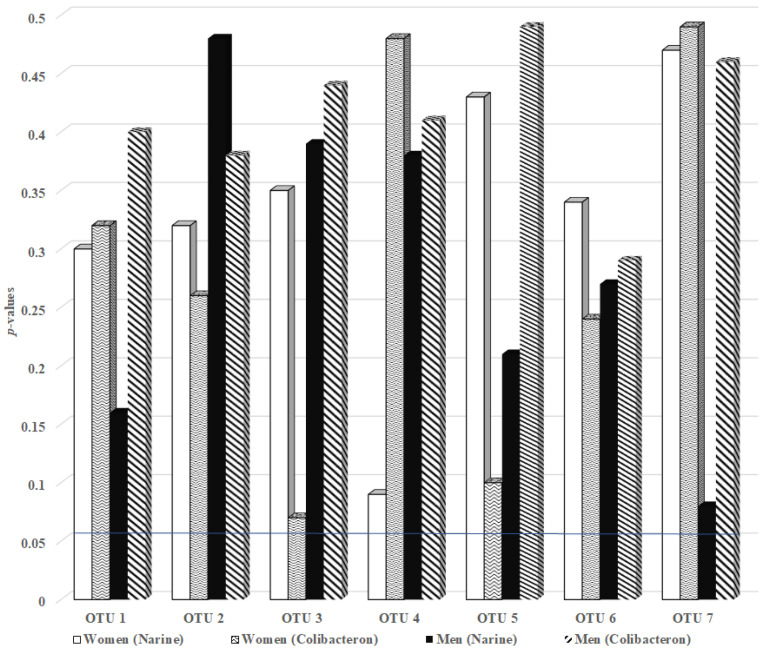
*p*-values for changes in hybridization scores of gut *Candidatus saccharibacteria* spp. (formerly TM7) before and after administration of Narine and Colibacteron probiotics in FMF patients. Hybridization scores are presented as mean ± standard deviation; OTU—operational taxonomic unit.

The administration of Narine and Colibacteron probiotics did not result in statistically significant changes in the hybridization scores for *Candidatus saccharibacteria* spp. in either female or male FMF patients (all *p* > 0.05, Figure 1).

In female patients, the lowest *p*-values were observed for OTU 4 after Narine intake (*p* = 0.09) and for OTU 3 following Colibacteron administration (*p* = 0.07), while in male patients, OTU 7 showed the lowest *p*-value after Narine (*p* = 0.08). For all other OTUs *p*-values ranged from 0.10 to 0.49, indicating no significant differences between pre- and post-intervention levels.

Given the low *p*-values observed for certain OTUs (e.g., OTU 3 in women after Colibacteron, *p* = 0.07; OTU 4 in women after Narine, *p* = 0.09), and considering the modest sample size (n = 15 per subgroup), it is plausible that the lack of statistical significance may reflect insufficient statistical power rather than a true absence of effect. Under conditions with larger cohorts or extended intervention duration, these trends may reach significance, suggesting a potential modulatory capacity of both probiotics on saccharibacteria populations.

### 2.3. Gut Schaalia odontolytica Distribution in FMF-Affected and Healthy Women

The hybridization scores of *Schaalia odontolytica* OTUs before and after placebo administration in healthy and FMF-affected women are presented in Table 2.

Overall, the hybridization signals were low in both groups, suggesting that *S. odontolytica* is present in the gut at low abundance. Four OTUs assigned to *S. odontolytica* were analyzed.

Among healthy women some OTUs exhibited a noticeable decrease in hybridization intensity following placebo administration. In particular, OUT 3 showed a statistically significant reduction (*p* = 0.048), while OTU 2 and OTU 4 approached significance (*p* = 0.054 and *p* = 0.057, respectively). Additionally, OTU 1 remained stable (*p* = 0.40).

In FMF-affected women no significant shifts were observed after placebo administration, with *p* values ranging from 0.27 to 0.38 for all four OTUs.

These observations suggest that the gut *S. odontolytica* population in FMF patients may be less responsive to placebo, though low hybridization scores and the modest sample size (n = 15 per group) warrant cautious interpretation. Although the placebo consisted only of an inert gelatin capsule, some OTUs, particularly in healthy women, exhibited notable shifts, including one statistically significant decrease (OTU 3, *p* = 0.048). These findings may reflect individual-level variability in microbiota composition and responsiveness, especially given the modest sample size (n = 15). However, it is also plausible that placebo-related factors, such as expectation-induced changes or gut–brain axis dynamics, played a role. Previous studies have shown that even inert substances can modulate gut microbial signatures via neuroimmune pathways, particularly in populations with heightened sensitivity or under stress [20]. These results therefore underscore the importance of including placebo controls in microbiota intervention studies and suggest that host–microbe interactions may respond to perceived interventions even in the absence of active compounds.

### 2.4. Gut Schaalia odontolytica Distribution in FMF-Affected and Healthy Men

The hybridization scores of *Schaalia odontolytica* OTUs in healthy and FMF-affected men before and after placebo administration are presented in Table 3.

As with women, overall hybridization signals were low in all male subgroups, indicating a low relative abundance of *S. odontolytica* in the gut microbiota.

Across all OTUs (OTU 1–OTU 4) no statistically significant differences were observed between pre- and post-placebo values (*p* > 0.05) in either group. For instance, OTU 1 in healthy men increased from 4033 ± 2110 to 5551 ± 1881 after placebo, yet the change was not significant (*p* = 0.16). The remaining OTUs also showed no significant alterations, with *p* values ranging from 0.37 to 0.49. Similarly, in FMF-affected men no OTU demonstrated a significant response to placebo administration (all *p* values exceeded 0.05). These findings suggest a general stability of *S. odontolytica* OTUs in the male gut microbiome regardless of FMF status, though the low hybridization signals and modest sample size (n = 15 per group) limit definitive conclusions.

### 2.5. The Effect of Probiotic Narine and Colibacteron on the Gut Schaalia odontolytica OTUs in FMF-Affected Men and Women

The hybridization scores of *S. odontolytica* OTUs in FMF patients following the administration of the probiotics Narine and Colibacteron are presented in Table 4.

Overall, no statistically significant changes were observed across the four OTUs in either sex after probiotic intervention (*p* > 0.05).

In FMF women, administration of Narine was associated with a trend toward increased hybridization scores in the following three out of four OTUs:

- OTU 2 increased from 1975.00 ± 1135.00 to 4611.00 ± 2725.00 (*p* = 0.063),
- OTU 3 from 2098.00 ± 788.00 to 2835.00 ± 1477.00 (*p* = 0.081),
- OTU 4 from 2285.00 ± 695.00 to 5480.00 ± 2944.00 (*p* = 0.074).

Although these values did not meet the conventional threshold for significance, the upward trends may suggest a potential modulatory effect of Narine on the abundance of specific *S. odontolytica* OTUs in FMF women.

Conversely, administration of Colibacteron in FMF women was associated with a downward trend in OTU 3, with scores decreasing from 2614.00 ± 953.00 to 1803.00 ± 631.00 (*p* = 0.08).

In FMF men, no noticeable trends were observed for either probiotic. All *p*-values remained well above 0.05, indicating a lack of probiotic-associated modulation in this group. However, due to the low hybridization signals and limited sample size (n = 15 per subgroup) these observations should be interpreted with caution.

## 3. Discussion

FMF exhibits higher prevalence among males in the Armenian population [21,22,23], highlighting the importance of investigating sex-based differences in disease presentation and gut microbiota composition [24].

The clinical picture of FMF in men can be more severe. Skin inflammation, serositis, and in some cases, testicular inflammation are often observed. They are also considered to be at higher risk of developing serum amyloidosis, especially in the case of homozygous manifestation of the M694V mutation [25,26].

In women, FMF is often accompanied by an intensity of symptoms caused by hormonal changes. Pain may worsen during certain phases of the menstrual cycle. In addition, women sometimes experience complications during conception and pregnancy, but with appropriate treatment complications are preventable [27,28].

In addition to saccharibacterial shifts, Narine administration has been shown to modulate other gut microbes—including reductions in Enterobacteriaceae, modulation of *Enterococcus faecalis*, and stable *Lactobacillus* populations—demonstrating systemic probiotic effects that occurred parallel to those reported here [29]. Prior research has documented sex-specific variations in depression indices and gut–brain axis dynamics in FMF patients [20], too. Extending this line of inquiry, current studies explore the sex-dependent patterns of *Candidatus saccharibacteria* spp. and *Schaalia odontolytica* OTUs within the gut microbiota of both healthy controls and FMF patients using PhyloChip™ microarray analysis.

### 3.1. Potential Association Between Candidatus saccharibacteria spp. and Schaalia odontolytica in Healthy Individuals and FMF Patients: Trends Observed Under Placebo Conditions

Placebo studies (using inert substances) are crucial for nutritional and clinical research, offering insights into treatment expectations and highlighting the role of the gut–brain axis in modulating physiological responses [30,31]. Our previous studies showed a decrease in *Candidatus saccharibacteria* OTUs in the group of healthy women receiving placebo [9]. In the current study, a decrease in gut *Schaalia odontolytica* OTUs was also observed in healthy women (Table 2). In healthy men, placebo treatment did not cause notable changes in either *Candidatus saccharibacteria* spp. (Table 1) or *Schaalia odontolytica* OTUs (Table 3). A similar trend was noted in the placebo subgroups of FMF men (Table 1 and Table 3) and FMF women [9] (Table 2).

Although these findings hint at a parallel behavior between these two taxa, the small sample size and lack of consistent statistical significance limit the strength of any firm conclusions. It is therefore premature to assert a biologically significant association. However, the observed co-fluctuation in healthy women raises the hypothesis that *Candidatus saccharibacteria* spp. and *S. odontolytica* might share certain ecological niches or interaction patterns. Possible mechanisms that could explain this trend include shared mucosal microenvironment, quorum sensing and co-regulated growth [32,33], metabolic cooperation or dependency, structural biofilm interactions [34], and host-driven constraints in FMF, as further discussed in the Supplementary Materials.

In summary, the observed co-behavior of *Candidatus saccharibacteria* and *S. odontolytica* in healthy placebo-treated women could represent a microbiological phenomenon worth further exploration. Future metagenomic, transcriptomic, and mechanistic studies are needed to test whether these taxa functionally interact or simply fluctuate in parallel under shared conditions.

### 3.2. Differential Response of Candidatus saccharibacteria and Schaalia odontolytica to Narine Administration in FMF Patients Relative to Placebo

The global probiotic market continues to expand, driven by increasing interest in both agricultural [35,36] and healthcare [37] contexts [22,38]. According to Pepoyan et al. [29], administration of *L. acidophilus* INMIA 9602 in male FMF patients led to a significant reduction in *Candida albicans* prevalence and a marked decrease in Enterobacteriaceae abundance, independent of *C. albicans* carrier status. Additionally, Narine exerted sex-dependent effects on *Enterococcus faecalis*, decreasing its abundance in all female FMF patients and selectively increasing in males. Systemically, Narine normalized elevated C-reactive protein and erythrocyte sedimentation rate levels in FMF patients [22].

Under placebo conditions neither *Candidatus saccharibacteria* nor *Schaalia odontolytica* exhibited statistically significant changes in OTU hybridization scores for FMF patients of either sex (all *p* > 0.05). Both taxa remained essentially stable, demonstrating that minor fluctuations did not translate into meaningful shifts in the absence of active intervention. Narine administration elicited directional, sex-specific changes, suggesting a potential modulatory effect beyond random or expectancy-driven variation. Several non-mutually exclusive mechanisms may explain Narine’s selective influence on these taxa against a stable placebo backdrop, such as lactic acid-mediated pH shifts [39,40], quorum-sensing interference [41], immune modulation and barrier restoration [42,43,44], metabolic cross-feeding loops, and differential colonization resistance, as further elaborated in the Supplementary Materials. Healthy individuals often exhibit robust colonization resistance, whereas FMF patients experience reduced microbial diversity due to chronic inflammation and colchicine’s effects on epithelial microtubule function. As a result, Narine may establish more readily in FMF guts, selectively altering niche availability. In FMF women, Narine may outcompete specific competitors, freeing ecological niches for *Candidatus saccharibacteria*. In FMF men, competition dynamics may differ, allowing *S. odontolytica* to flourish when Narine improves overall barrier function and nutrient availability.

However, the clinical or biological desirability of these shifts remains uncertain. *Candidatus saccharibacteria* is primarily characterized through environmental and oral studies, and its gut functions, if any, are not well defined. Similarly, *S. odontolytica* is traditionally viewed as an oral commensal or opportunistic pathogen. In the absence of data linking changes in their abundance to host benefit or harm, the observed modulation by Narine may indicate microbiome-level effects but cannot yet be interpreted as therapeutic.

Thus, under placebo conditions both *Candidatus saccharibacteria* and *Schaalia odontolytica* remained essentially unchanged in FMF patients. The directional, sex-dependent shifts observed only with Narine administration—particularly the increase in *Candidatus saccharibacteria* OTU 4 in FMF women and the trend toward increased *S. odontolytica* OTUs in FMF men—may indicate that Narine exerts a potentially biologically relevant influence beyond placebo. These findings suggest a possible selective modulation of CPR and actinobacterial populations by Narine in FMF patients. Further mechanistic studies (e.g., metatranscriptomic profiling, in vitro coculture experiments) are needed to confirm and characterize the pathways proposed above.

### 3.3. Differential Response of Candidatus saccharibacteria and Schaalia odontolytica to Colibacteron Administration in FMF Patients Relative to Placebo

Under placebo administration both *Candidatus saccharibacteria* and *Schaalia odontolytica* OTU hybridization scores remained statistically unchanged in FMF patients of either sex (all *p* > 0.05). These stable baseline profiles confirm that, in the absence of active probiotic intervention, neither taxon exhibits meaningful fluctuations. In contrast, Colibacteron intervention produced directional taxon- and sex-specific trends that were not replicated under placebo, indicating a bona fide modulatory effect.

Collectively, these sex-stratified patterns demonstrate that Colibacteron’s modulatory effects are confined primarily to FMF women, where specific OTUs of both *Candidatus saccharibacteria* and *S. odontolytica* show downward trends, while FMF men exhibit no analogous responses. The lack of any placebo-driven declines in those same OTUs reinforces Colibacteron’s genuine, targeted action. Potential mechanisms underlying these selective effects, such as metabolic competition, immune modulation, and niche-specific interactions, are discussed in detail in the Supplementary Materials.

Thus, under placebo conditions both *Candidatus saccharibacteria* and *S. odontolytica* remained stable in FMF patients. Colibacteron administration, however, induced sex-specific downward trends, particularly in OTU 3 populations of FMF women, that were absent under placebo. These observations may indicate a potential biological activity of Colibacteron against select CPR and actinobacterial subpopulations in FMF. The lack of comparable shifts in FMF men suggests that Colibacteron’s modulatory effects might be constrained by sex-specific mucosal, immunological, and metabolic contexts. Future mechanistic studies (e.g., targeted metabolomics, epithelial glycosylation assays, in vitro biofilm disruption models) are required to validate and further explore the pathways hypothesized above.

Notably, reductions in *Candida albicans* and enterobacterial abundance observed in Narine-treated FMF patients were paralleled by the normalization of elevated C-reactive protein (CRP) levels in a subset of participants [29]; this provides preliminary clinical context to the microbial findings.

## 4. Materials and Methods

### 4.1. Study Population

The methodology employed in this study is based on previously published protocols [45,46,47,48]. The complete datasets are available in the NCBI Gene Expression Omnibus (GEO; GSE111835; https://www.ncbi.nlm.nih.gov/geo/query/acc.cgi?acc=GSE111835, accessed on 16 March 2008; last modified 16 July 2018). Ethical approval was granted by the Ethics Committee of the Armenian National Agrarian University and the Higher Education and Science Committee of the Republic of Armenia (Protocol No. 10-15-21AG, dated 21 October 2021).

The study population included male and female patients diagnosed with FMF who had been receiving colchicine treatment for more than seven years. Given the established impact of host genetics [24,49], sex [50,51], age [52,53], health status [54,55], dietary habits [56,57,58,59], and various environmental factors [60,61,62,63] on the gut microbiota, the study cohort included both FMF patients homozygous for the M694V mutation and healthy Armenian adults residing in Yerevan.

A total of 60 participants were enrolled, 30 FMF patients and 30 non-FMF controls, with an equal male-to-female ratio (1:1). Participant selection was based on age (18–45 years) and test results from clinical, biochemical, immunological, and genetic blood analyses.

### 4.2. Sample Collection and Analysis

Stool samples were collected at two time points: before the initiation of any treatment and after a one-month course of twice-daily probiotic or placebo administration.

All samples were promptly refrigerated and subsequently processed for microbial community analysis using a third-generation, high-density DNA-microarray, PhyloChip™ (Affymetrix, Santa Clara, CA, USA). This culture-independent approach, as previously described by Kellogg et al. [64] and Pepoyan et al. [29], enables the detection and abundance estimation of bacterial diversity through variations in hybridization signal intensity. In the current study, 18,723 operational taxonomic units (OTUs) were identified, including 7 OTUs affiliated with *Candidatus saccharibacteria* spp. and 4 OTUs corresponding to *Schaalia odontolytica*. Notably, all 4 OTUs assigned to *S. odontolytica* (based on probe-level hybridization profiles from the PhyloChip™ data) matched reference genomes annotated as *S. odontolytica*, although they represent different OTU IDs. This suggests they likely correspond to strain-level or sequence-variant diversity within a single species rather than distinct species. In contrast, the 7 OTUs related to *Candidatus saccharibacteria* belong to unresolved lineages without species-level annotation, hence the use of “spp.” to reflect that taxonomic uncertainty. Additionally, due to the perfect match/mismatch probe system used in PhyloChip™ analysis low-abundance OTUs such as those representing CPR taxa can be robustly detected, ensuring reliable profiling of these underrepresented microbial groups.

### 4.3. Intervention and Controls

A partially blinded, placebo-controlled clinical trial was conducted using commercial preparations produced by VITAMAX-E (Armenia). These included the “Narine” preparation containing *Lactobacillus acidophilus* INMIA 9602 and the “Colibacteron” preparation containing *Escherichia coli* M-17, as well as empty gelatin placebo capsules. FMF and non-FMF individuals received either a probiotic or placebo to assess changes in gut microbiota before and after intervention.

During the study participants avoided all dietary supplements and fermented foods except for the tested probiotics.

### 4.4. DNA Extraction and PhyloChip Analysis

DNA was extracted from fecal samples stored at −80 °C using two commercial kits in parallel—ZR Fecal DNA MiniPrep™ (Zymo Research Corp., Irvine, CA, USA) and UltraClean^®^ Fecal DNA Isolation Kit (MoBio Laboratories Inc., Carlsbad, CA, USA)—in order to minimize DNA loss. The concentration and purity of the extracted DNA were evaluated spectrophotometrically (NanoDrop, Thermo Fisher Scientific, Waltham, MA, USA).

The nearly full-length 16S rRNA gene fragments were amplified using the universal bacterial primers 27f.jgi and 1492r.jgi, originally developed by the Joint Genome Institute (JGI; https://jgi.doe.gov, accessed on 11 November 2024), which are specific for bacterial and bacterial/archaeal domains, respectively.

The amplified 16S rRNA products were then hybridized to the PhyloChip™ DNA-microarray. This platform contains over one million oligonucleotide probes and enables the detection of more than 50,000 bacterial taxa by measuring hybridization intensities. Microbial diversity and relative abundance were inferred from these intensity values. Presence/absence calls were based on predefined thresholds of probe-set signals, while relative abundance was estimated by summing fluorescence intensities of probes corresponding to each OTU, following the approach described by Kellogg et al. [64] and Pepoyan et al. [29].

A total of 7 operational taxonomic units (OTUs) were assigned to *Candidatus saccharibacteria* spp. and 4 OTUs to *Schaalia odontolytica*, based on hybridization patterns across multiple probe sets. Taxonomic assignment was conducted using the PhyloChip™ analysis pipeline, in which each OTU is defined by a cluster of perfect-match probes targeting specific regions of the 16S rRNA gene. These OTUs reflect sequence-level diversity within each taxonomic group and may correspond to different strains or subpopulations. Only OTUs that met stringent hybridization and specificity criteria (i.e., multiple high-intensity probe signals and consistent presence across replicates) were included in the final analysis.

This manuscript underwent professional language and style editing with the assistance of ChatGPT (https://chat.openai.com; accessed on 15 June 2025, OpenAI, San Francisco, CA, USA).

## 5. Conclusions

This study identified sex-dependent trends in the gut abundance of *Candidatus saccharibacteria* and *Schaalia odontolytica* in Armenian FMF patients, with limited but suggestive responses to probiotic and placebo interventions. Most taxa remained stable under placebo, reinforcing the importance of placebo-controlled baselines for interpreting microbial shifts. While most changes did not reach statistical significance, probiotic-specific trends, particularly in FMF women, indicated potential sex-specific effects. These may involve mechanisms such as immune modulation, pH changes, or biofilm disruption, though confirmation requires larger, multi-omics studies.

Given the known roles of these taxa in oral biofilms, gut-targeted probiotics might also influence oral microbiota, a possibility warranting further exploration. Future research integrating host response data and oral microbiome profiling could clarify the functional relevance of these microbial dynamics and support the development of sex-informed microbiota-based therapies.

While the clinical relevance of *Candidatus saccharibacteria* and *Schaalia odontolytica* fluctuations remains speculative, these taxa may participate in broader host–microbiota dynamics, including immune modulation, mucosal interactions, and microbial competition. Clarifying their functional roles requires dedicated mechanistic studies and could reveal new microbiota-based therapeutic targets.

A formal power calculation should also be included in future studies to ensure adequate statistical power for detecting subtle microbial shifts.

## Figures and Tables

**Table 1:** Hybridization scores of gut *Candidatus saccharibacteria* spp. (formerly TM7) in healthy and FMF-affected males before and after placebo administration (mean ± SD).

OTU	Healthy Men Before	Healthy Men After	*p* (Healthy)	FMF Men Before	FMF Men After	*p* (FMF)	*p* (Healthy vs. FMF)
OTU 1	4870.98 ± 1117.07	5363.95 ± 727.29	0.39	6099.00 ± 1429.00	5816.00.00 ± 2018	0.36	>0.05
OTU 2	373.53 ± 40.42	462.18 ± 173.55	0.28	943.00 ± 699.00	1173.00 ± 769.00	0.25	>0.05
OTU 3	573.04 ± 635.78	369.85 ± 212.22	0.61	688.00 ± 1373.00	653.00 ± 741.00	0.47	>0.05
OTU 4	4094.13 ± 1261.68	4717.18 ± 392.56	0.44	3852.00 ± 1989.00	5120.00 ± 2089.00	0.09	>0.05
OTU 5	396.35 ± 97.72	422.86 ± 212.87	0.71	460.00 ± 361.00	457.00 ± 145.00	0.49	>0.05
OTU 6	4026.52 ± 1735.20	3478.66 ± 735.08	0.58	3912.00 ± 1999.00	4046.00 ± 1448.00	0.43	>0.05
OTU 7	2768.55 ± 1718.45	3202.88 ± 1481.22	0.64	3081.00 ± 948.00	3017.00 ± 1362.00	0.45	>0.05

Note: FMF—familial mediterranean fever. *p*—*p* value indicating statistical significance of differences in hybridization scores before and after placebo administration, and between healthy and FMF males. *p* < 0.05 is considered statistically significant.

**Table 2:** The differences in hybridization scores of gut *Schaalia odontolytica* before and after placebo administration in healthy and FMF women.

OTUs	Hybridization Scores					
	Healthy Women			FMF Women		
	Before the Placebo Administration	After the Placebo Administration	*p* Before/After Placebo Administration	After the Placebo Administration	Before the Placebo Administration	*p* Before/After Placebo Administration
OTU 1	5089.00 ± 2419.00	4854.00 ± 1620.00	0.400	3804.00 ± 619.00	3655.00 ± 870.00	0.38
OTU 2	3484.00 ± 3325.00	2019.00 ± 1128.00	0.054	4051.00 ± 2751.00	5063.00 ± 2811.00	0.29
OTU 3	2185.00 ± 1305.00	1457.00 ± 637.00	0.048	2216.00 ± 1034.00	2523.00 ± 976.00	0.32
OTU 4	3494.00 ± 3711.00	1836.00 ± 1136.00	0.057	3802.00 ± 2865.00	4959.00 ± 2990.00	0.27

Note: Hybridization scores are presented as mean ± standard deviation.

**Table 3:** Hybridization scores of gut *Schaalia odontolytica* OTUs before and after placebo administration in healthy and FMF-affected men.

OTU	Hybridization Scores (Mean ± SD).					
	Healthy Men			FMF Men		
	Before the Placebo Administration	After the Placebo Administration	*p* Before/After Placebo Administration	After the Placebo Administration	Before the Placebo Administration	*p* Before/After Placebo Administration
OTU 1	4033.00 ± 2110.00	5551.40 ± 1880.57	0.16	3234.00 ± 1075.00	3580.00 ± 1461.00	0.28
OTU 2	2749.00 ± 1118.00	2609.87 ± 953.00	0.43	3311.00 ± 1759.00	3917.00 ± 2158.00	0.25
OTU 3	2034.00 ± 887.00	2015.68 ± 779.50	0.49	2202.00 ± 623.00	2540.00 ± 691.00	0.13
OTU 4	2502.00 ± 1123.00	2275.89 ± 687.91	0.37	3154.00 ± 1589.00	3725.00 ± 2327.00	0.26

**Table 4:** *p*-values for changes in hybridization scores of gut *Schaalia odontolytica* OTUs before and after administration of Narine and Colibacteron in FMF patients.

OTU	FMF Women		FMF Men	
	Narine	Colibacteron	Narine	Colibacteron
OTU 1	0.352	0.290	0.490	0.190
OTU 2	0.063	0.170	0.470	0.310
OTU 3	0.081	0.080	0.490	0.340
OTU 4	0.074	0.130	0.390	0.390

Note: Changes in OTU hybridization scores were evaluated using paired comparisons before and after the probiotic treatment.

## Data Availability

Data is contained within the article and Supplementary Materials.

## Supplementary Materials

The following supporting information can be downloaded at: https://www.mdpi.com/article/10.3390/ijms26188959/s1.

## Abbreviations

The following abbreviations are used in this manuscript:

CPR	Candidate Phyla Radiation
FMF	Familial Mediterranean fever
OTU	Operational Taxonomic Unit
SCFA	Short-Chain Fatty Acid
LPS	Lipopolysaccharide
IL	Interleukin
QS	Quorum Sensing
