# Supplementary material for: Preliminary Evidence for Sex-Specific Trends in Probiotic Modulation of Gut Saccharibacteria in Familial Mediterranean Fever Patients: Effects of Lactobacillus acidophilus INMIA 9602 Er 317/402 and Escherichia coli M-17
Source: Int J Mol Sci. 2025 Sep 15;26(18):8959. doi: 10.3390/ijms26188959 (PMC12469711; doi:10.3390/ijms26188959)
Supplement: Supplementary file 1 [file ijms-26-08959-s001.zip › ijms-3755572-supplementary.pdf]

Section 3.1

Potential association between Candidatus Saccharibacteria spp. and Schaalia odontolytica in healthy individuals and FMF patients: trends observed under placebo conditions

Placebo studies (using inert substances) are crucial for nutritional and clinical research, offering insights into treatment expectations and highlighting the role of the gut–brain axis in modulating physiological responses [30,31]. Our previous studies showed a decrease in *Candidatus Saccharibacteria* OTUs in the group of healthy women receiving placebo [9]. In the current study, a decrease in gut *Schaalia odontolytica* OTUs was also observed in healthy women (Table 2). In healthy men, placebo treatment did not cause notable changes in either *Candidatus Saccharibacteria* spp. (Table 1) or *Schaalia odontolytica* OTUs (Table 3). A similar trend was noted in the placebo subgroups of FMF men (Table 1 and Table 3) and FMF women [9], (Table 2).

Although these findings hint at a parallel behavior between these two taxa, the small sample size and lack of consistent statistical significance limit the strength of any firm conclusions. It is therefore premature to assert a biologically significant association. However, the observed co-fluctuation in healthy women raises the hypothesis that *Candidatus Saccharibacteria* spp. and *S. odontolytica* might share certain ecological niches or interaction patterns.

Possible mechanisms that could explain this trend include:

• Shared mucosal microenvironment. Both taxa may respond similarly to changes in mucosal biochemical conditions, such as fluctuations in pH, host-derived immune factors, or nutrient availability.

• Quorum sensing and co-regulated growth [32]. *S. odontolytica* might engage in autoinducer-2 (AI-2)–based quorum sensing, potentially facilitating the growth of epibiotic taxa like *Candidatus Saccharibacteria* [33].

• Metabolic cooperation or dependency. *Candidatus Saccharibacteria* spp., with their reduced genomes, may rely on co-occurring bacteria (such as *S. odontolytica*) for essential metabolites [33].

• Structural biofilm interactions. These taxa may interact physically in biofilms, indicating niche co-adaptation or consortium-level cooperation [34].

• Host-driven constraints in FMF. Chronic inflammation or innate immune dysregulation in FMF (e.g., pyrin inflammasome activity) may dampen microbial plasticity, affecting co-dynamics.

Section 3.2.

Differential response of Candidatus Saccharibacteria and Schaalia odontolytica to Narine administration in FMF patients relative to placebo

The global probiotic market continues to expand, driven by increasing interest in both agricultural [35,36] and healthcare [37] contexts [22,38]. According to Pepoyan *et al.* [29], administration of *L. acidophilus* INMIA 9602 in male FMF patients led to a significant reduction in *Candida albicans* prevalence and a marked decrease in *Enterobacteriaceae* abundance, independent of *C. albicans* carrier status. Additionally, Narine exerted sex-dependent effects on *Enterococcus faecalis*, decreasing its abundance in all female FMF patients and selectively increasing in males. Systemically, Narine normalized elevated C-reactive protein and erythrocyte sedimentation rate levels in FMF patients [22].

Under placebo conditions, neither *Candidatus Saccharibacteria* nor *Schaalia odontolytica* exhibited statistically significant changes in OTU hybridization scores for FMF patients of either sex (all $p > 0.05$). Both taxa remained essentially stable, demonstrating that minor fluctuations did not translate into meaningful shifts

in the absence of active intervention. Narine administration elicited directional, sex-specific changes, suggesting a potential modulatory effect beyond random or expectancy-driven variation.

- FMF women:
  - *Candidatus Saccharibacteria* OTU 4 exhibited a pronounced upward trend after Narine intake ($p \approx 0.074$), whereas placebo had produced no significant change ($p > 0.05$).
  - *Schaalia odontolytica* OTUs 2 and 4 increased in hybridization intensity following Narine ($p \approx 0.063$ and $p \approx 0.074$, respectively), despite placebo failing to induce any such directionality. These parallel rises in OTU abundance under Narine, but not placebo, underscore a targeted probiotic effect on both the CPR bacterial taxon (*Candidatus Saccharibacteria*) and its putative actinobacterial host (*S. odontolytica*).
- FMF men:
  - No *Candidatus Saccharibacteria* OTUs demonstrated statistically meaningful shifts under Narine (all $p > 0.10$).
  - *Schaalia odontolytica* OTU 2 displayed a non-significant upward tendency with Narine ($p \approx 0.081$), not replicated by placebo.

Collectively, these data suggest that Narine primarily modulates *S. odontolytica* in FMF men while exerting a stronger influence on *Candidatus Saccharibacteria* in FMF women.

Several non-mutually exclusive mechanisms may explain Narine's selective influence on these taxa against a stable placebo backdrop:

- Lactic acid–mediated pH shifts. The strain of Narine ferments dietary carbohydrates into lactic acid [39]. FMF patients often experience chronic, low-grade inflammation and receive colchicine therapy, both of which can compromise mucosal barrier integrity. Localized acidification of the lumen by Narine can differentially affect CPR taxa and actinobacteria. *Candidatus Saccharibacteria* spp. typically thrives in slightly acidic microenvironments [40], whereas *S. odontolytica* may prefer neutral to mildly acidic pH. The Narine-induced pH drop can thereby favor *Candidatus Saccharibacteria* expansion in FMF women, while in FMF men, the combined inflammatory milieu and sex-specific mucosal glycosylation patterns may bias the response toward *S. odontolytica* proliferation.
- Quorum-sensing interference. *S. odontolytica* produces AI-2 and expresses QS genes such as *lsrB* and *luxS*, facilitating biofilm formation and interspecies communication [41]. Narine may secrete bacteriocins or signaling analogs that interfere with AI-2 signaling. In FMF contexts, where epithelial turnover is often altered, disruption of *S. odontolytica*'s QS network can indirectly affect its epibiotic partner, *Candidatus Saccharibacteria*, which relies on intimate biofilm associations to survive. The observed rise in *Candidatus Saccharibacteria* OTU 4 in FMF women may thus reflect enhanced AI-2 signaling (or reduced QS inhibition) in the presence of *L. acidophilus*-derived mediators, whereas in FMF men, Narine's impact on QS could favor *S. odontolytica* expansion.
- Immune modulation and barrier restoration. FMF's hallmark pyrin inflammasome hyperactivation generates chronic, subclinical inflammation that disrupts epithelial tight junctions. Lactobacilli are known to upregulate mucin production and strengthen tight junction proteins (e.g., occluding [42,43], claudins [44]. In FMF women, whose hormonal milieu further modulates mucosal glycosylation, Narine may restore barrier integrity more effectively, creating microhabitats conducive to *Candidatus Saccharibacteria*. Conversely, in FMF men, the baseline epithelial environment might favor *S. odontolytica* once Narine normalizes local immune signaling (for instance, by inducing IL-10 production and suppressing IL-1β). Therefore, sex-dependent immune responses to Narine likely underlie the asymmetrical OTU shifts.
- Metabolic cross-feeding loops**.** Both CPR taxa and actinobacteria participate in specialized metabolic consortia. Narine may enhance the production of short-chain fatty acids (SCFAs), particularly acetate

and lactate, which serve as substrates for butyrate-producing Firmicutes. In FMF women, a reduction in inflammation, coupled with increased SCFA availability, may indirectly promote the growth of Candidatus Saccharibacteria, as CPR members often rely on fermentation by-products produced by neighboring microbes. In FMF men, SCFA dynamics may instead favor the saccharolytic metabolism of S. odontolytica. These cross-feeding interactions likely become more pronounced under dysbiotic FMF conditions, which may explain why placebo fails to induce similar metabolic cascades.

- Differential colonization resistance. Healthy individuals often exhibit robust colonization resistance, whereas FMF patients experience reduced microbial diversity due to chronic inflammation and colchicine's effects on epithelial microtubule function. As a result, Narine may establish more readily in FMF guts, selectively altering niche availability. In FMF women, Narine may outcompete specific competitors, freeing ecological niches for *Candidatus Saccharibacteria*. In FMF men, competition dynamics may differ, allowing *S. odontolytica* to flourish when Narine improves overall barrier function and nutrient availability.

However, the clinical or biological desirability of these shifts remains uncertain. *Candidatus Saccharibacteria* is primarily characterized through environmental and oral studies, and its gut functions, if anyare not well defined. Similarly, *S. odontolytica* is traditionally viewed as an oral commensal or opportunistic pathogen. In the absence of data linking changes in their abundance to host benefit or harm, the observed modulation by Narine may indicate microbiome-level effects, but cannot yet be interpreted as therapeutic.

Section 3.3.

Differential response of Candidatus Saccharibacteria and Schaalia odontolytica to Colibacteron administration in FMF patients relative to placebo

Under placebo administration, both *Candidatus Saccharibacteria* and *Schaalia odontolytica* OTU hybridization scores remained statistically unchanged in FMF patients of either sex (all $p > 0.05$). These stable baseline profiles confirm that, in the absence of active probiotic intervention, neither taxon exhibits meaningful fluctuations. By contrast, Colibacteron intervention produced directional, taxon- and sex-specific trends that were not replicated under placebo, indicating a bona fide modulatory effect.

- FMF women
- *Candidatus Saccharibacteria*: None of the seven OTUs demonstrated statistically significant shifts following Colibacteron (all $p \geq 0.07$). However, OTU 3 exhibited a downward trend (mean decrease; $p \approx 0.07$), despite showing no change under placebo ($p > 0.05$). Similarly, OTU 5 showed a non-significant decline ($p \approx 0.10$) after Colibacteron, again contrasted with placebo's stability. These downward tendencies, absent in placebo, imply that Colibacteron exerts a subtle suppressive influence on certain *Candidatus Saccharibacteria* OTUs in FMF females.
- *Schaalia odontolytica*: In FMF women, OTU 3 decreased more sharply after Colibacteron (mean decline; $p \approx 0.08$), while placebo's effect on the same OTU was negligible ($p > 0.05$). OTUs 1, 2, and 4 remained statistically stable ($p > 0.10$) under Colibacteron, mirroring placebo outcomes. The specific downtrend in OTU 3, absent with placebo, suggests that Colibacteron targets this *S. odontolytica* subpopulation, potentially shifting community composition toward lower overall abundance.
- FMF men
- *Candidatus Saccharibacteria*: Across all seven OTUs, Colibacteron failed to elicit statistically significant changes in FMF men (all $p > 0.30$), and no directional trends appeared. Placebo administration likewise did not alter these OTUs (all $p > 0.05$). The absence of both Colibacteron-

and placebo-driven shifts indicates that Colibacteron's suppressive or stimulatory impact on *Candidatus Saccharibacteria* is minimal or overridden by other factors in FMF males.
- *Schaalia odontolytica*: For OTUs 1–4, Colibacteron produced non-significant changes (all $p > 0.30$) that did not deviate directionally from placebo. Both interventions yielded stable OTU scores in FMF men, suggesting that *S. odontolytica* is refractory to Colibacteron in this subgroup.

Collectively, these sex-stratified patterns demonstrate that Colibacteron's modulatory effects are confined primarily to FMF women, where specific OTUs of both *Candidatus Saccharibacteria* and *S. odontolytica* show downward trends, while FMF men exhibit no analogous responses. The lack of any placebo-driven declines in those same OTUs reinforces Colibacteron's genuine, targeted action.

Several non-mutually exclusive mechanisms may explain Colibacteron's selective influence on these taxa against a stable placebo backdrop:

- Competitive exclusion via *E. coli* M-17. Colibacteron contains *Escherichia coli* M-17, which may compete with CPR taxa for adhesion sites and simple carbohydrates. In FMF women, this competition could preferentially suppress *Candidatus Saccharibacteria* OTU 3 and *S. odontolytica* OTU 3, both of which may rely on similar mucosal niches or carbohydrate sources. In FMF men, however, higher baseline microbial diversity or distinct mucosal glycosylation patterns likely mitigate direct competition, explaining the absence of a Colibacteron effect.
- *LPS-Mediated immune signaling.* *E. coli* M-17 releases lipopolysaccharide (LPS), which can modulate innate immune responses. In FMF women, who may exhibit heightened mucosal immune sensitivity, LPS from Colibacteron could selectively enhance local production of antimicrobial peptides, disproportionately affecting OTU 3 of both *Candidatus Saccharibacteria* and *S. odontolytica*. FMF men's altered epithelial cytokine milieu might instead buffer this LPS effect, rendering CPR and ɯctinobacterial populations less susceptible.
- Biofilm disruption. *Candidatus Saccharibacteria* and *S. odontolytica* often co-localize within epibiotic biofilms. *E. coli* M-17 can secrete bacteriocins or SCFAs that disrupt biofilm architecture. In FMF women, biofilm integrity may already be compromised by chronic inflammation, making these taxa particularly vulnerable to Colibacteron-induced biofilm disruption. In FMF men, more robust biofilm formation could protect CPR and actinobacteria from similar perturbation.
- Modulation of mucosal lycosylation. FMF's systemic inflammation affects mucosal glycoprotein expression differently in women versus men. Colibacteron may further alter glycosylation patterns (e.g., fucosylation, sialylation) by inducing specific glycosyltransferase genes. These changes could decrease adherence sites for OTU 3 subpopulations in FMF women but have minimal effect in FMF men, where mucosal glycosylation remains less perturbed.
- SCFA shifts favoring alternative taxa. While Narine increases SCFA pools that benefit *Candidatus Saccharibacteria*, Colibacteron's *E. coli* metabolism may produce hydrogen and succinate, which inhibit CPR taxa. In FMF women, succinate accumulation could directly suppress OTU 3 of *Candidatus Saccharibacteria* and *S. odontolytica*, whereas FMF men's existing SCFA networks may neutralize succinate's suppressive action, preserving stable OTU profiles.
